# Case of primary intraocular lymphoproliferative disorder caused by Epstein–Barr Virus

**DOI:** 10.1186/s12886-020-01583-x

**Published:** 2020-07-28

**Authors:** Yumiko Ban, Masahiro Okamoto, Nahoko Ogata

**Affiliations:** Department of Ophthalmology, Nara Medical University, 840 Shijo-cho, Kashihara, Nara, 634-8521 Japan

**Keywords:** Epstein–Barr virus, Lymphoproliferative disorder, Masquerade syndrome, Uveitis, Flow cytometry

## Abstract

**Background:**

Cases of panuveitis caused by Epstein–Barr virus (EBV) associated with primary intraocular lymphoproliferative disorder (LPD) are rare in immunocompetent individuals.

**Case presentation:**

A 67-year-old man noted blurred vision in both eyes and was referred to our hospital. His best-corrected visual acuity (BCVA) was 20/20 in both eyes. He had mild inflammation in the anterior chamber but not in the vitreous of both eyes. The inflammation was resolved with topical corticosteroid but 10 months later both eyes presented recurrence. Treatment with a sub-Tenon’s injection of steroid was effective for OS but not for OD and 2 months after, the inflammation in the anterior chamber and vitreous opacities got worsen in OD and BCVA decreased to 6/20 OD. Thus, pars plana vitrectomy was performed on OD, and EBV was detected in the aqueous humor by multiplex polymerase chain reaction, and an infiltration of CD19κ positive B cells was revealed in the vitreous specimens by flow cytometry. Systemic workup revealed no other sites of lymphoproliferation, no active EBV infection, or underlying immunodeficiency.

**Conclusion:**

Panuveitis caused by EBV associated with primary intraocular LPD can occur in patients with no history of congenital or acquired immunodeficiencies.

## Background

Epstein–Barr virus (EBV), discovered in 1964 [1], has been associated with a number of malignancies [2]. Earlier studies have shown lymphoproliferative disorders (LPDs) in immunocompromised patients [3–5], but the number of patients who are immunocompetent have been very few. We report our findings in a case of panuveitis that was diagnosed as primary ocular LPD associated with EBV in an immunocompetent patient.

## Case report

A 67-year-old man with blurred vision in both eyes of one-month duration was referred to the Nara Medical University Hospital on January 19, 2016. His medical history showed that he had undergone cataract surgery on both eyes 7 years earlier, and he had also been diagnosed with chronic hepatitis C and interstitial pneumonia.

At his initial examination, his BCVA was 20/20 in both eyes, and his intraocular pressure (IOP) was 19 mmHg OD and 17 mmHg OS. He had + 2 inflammation according to SUN Workshop scale in the anterior chamber of the both eyes but not in the vitreous. Funduscopy showed no remarkable inflammatory findings in the retina and choroid. The inflammation of the anterior chamber in both eyes was resolved by 1% topical prednisolone acetate four times/day.

**Fig. 1:**
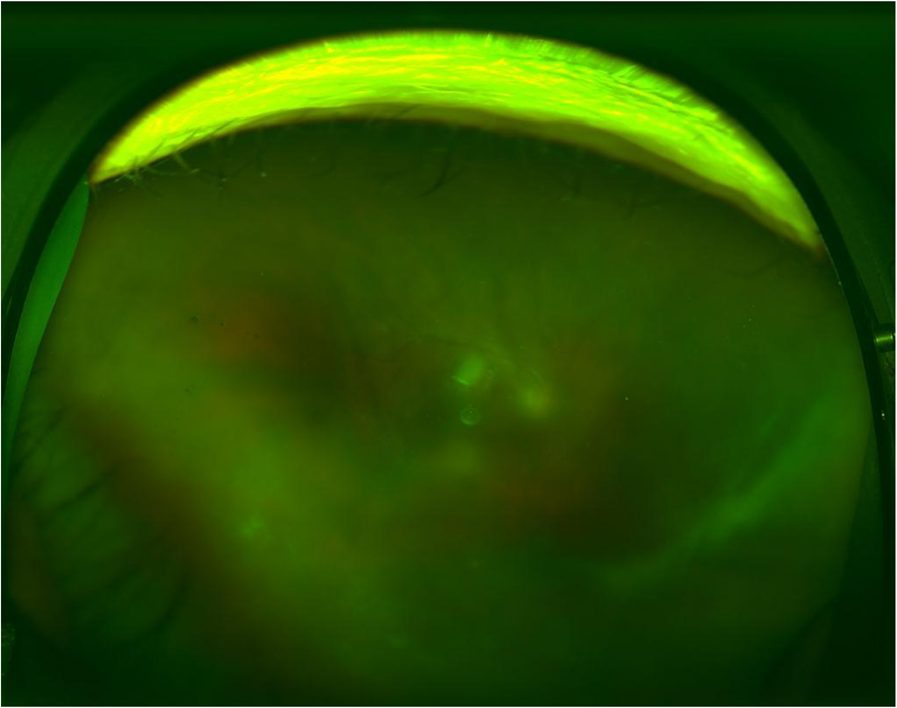
Preoperative fundus photographs of the right eye. This showed that the optic disc of the right eye appeared misty

Ten months after his initial visit, + 2 inflammations in the anterior chamber recurred with small white keratic precipitates, and + 1 inflammations vitreous opacities according to SUN Workshop scale were detected in both eyes. Topical prednisolone and sub-Tenon’s injection of triamcinolone acetonide were administrated for both eyes. These treatments were effective for the left eye but not for the right eye and 2 months after, the inflammation in the anterior chamber increased to + 3 and the opacities in the vitreous got worse to + 4 inflammation in the right eye (Fig. 1). His visual acuity in the right eye had decreased to 6/20 at this time. The results of laboratory examinations were essentially normal including blood cell counts, hepatic and renal function, serum levels of ACE, calcium, IL-2, and anti-HIV antibody, thus potential cause of immunosuppression was not detected.

A masquerade syndrome was suspected because the steroid therapy was not effective for the ocular inflammation. To confirm the diagnosis, 25-gauge microincision vitrectomy was performed and aqueous humor and undiluted vitreous samples were collected. During the operation, fine vitreous opacities were observed but the retina and choroid appeared to be normal otherwise.

**Fig. 2:**
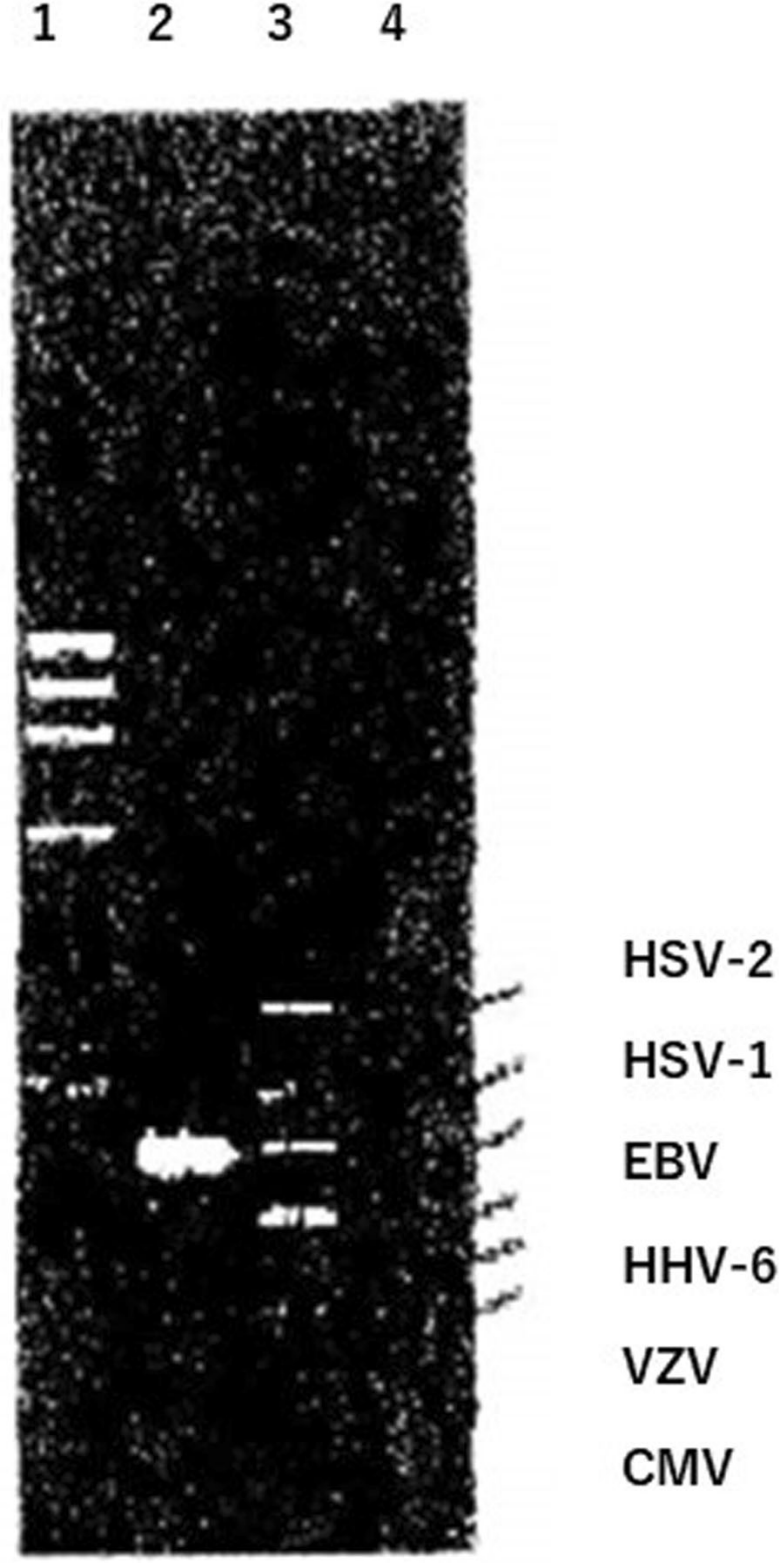
DNA for Epstein-Barr virus (EBV) was detected in the aqueous humor by multiplex polymerase chain reaction (PCR). 1 = size maker (φX174 DNA/HaeIII); 2 = patient sample; 3 = positive control; 4 = negative control

EBV was detected in the aqueous humor samples by multiplex polymerase chain reaction (PCR; Fig. 2). Flow cytometry of the vitreous samples showed CD3+ (T-cell marker) was 76.3%, CD19+ (B-cell marker) was 26.3%, CD19/κ was 45.5% and CD19/λ was 4.0%. These results indicated that the cells infiltrated in the vitreous were positive for B-cell and monoclonal increase of CD19/κ positive cells (Table 1). However, cytopathology from vitreous samples presented only lymphocytes with different kinds of maturity, plasma cells, and histocytes.

**Table 1:** Analysis of vitreous cells by flow cytometry

Specific Markers	Positive cells
CD3	76.3%
CD19	26.3%
CD19/κ	45.5%
CD19/λ	4.0%

The light chain ratio (LCR; κ chains % / λ chains %) is especially useful for diagnosing B-cell malignancies. Normal LCR is about 2. In this case, the κ and λ chains were 45.5 and 4.0%, respectively. Thus, the LCR was very high at about 11, suggesting that there is a high possibility of monoclonal B cell proliferation

Whole body examination by magnetic resonance imaging (MRI) and 2-Deoxy-2-[^18^F] fluoro-D-glucose positron emission tomography-computed tomography (FDG-PET/CT) were performed but no abnormal findings were found. Examinations of his peripheral blood samples showed that the antibody titer of EB virus capsid antigen (VCA) IgM was negative but the antibody titer of VCA-IgG was 640X (< 0.5 is negative). In addition, the titer of EBV-encoded nuclear antigen (EBNA) was 10X (< 0.5 is negative). These results indicated that he was an EBV carrier. The copy number of EBV DNA in the serum was below the detection limit (5 copies/μl), therefor a general chronic EBV infection was ruled out. Furthermore, an entire body examination showed that there were no systemic abnormalities and no immune deficiency was present.

**Fig. 3:**
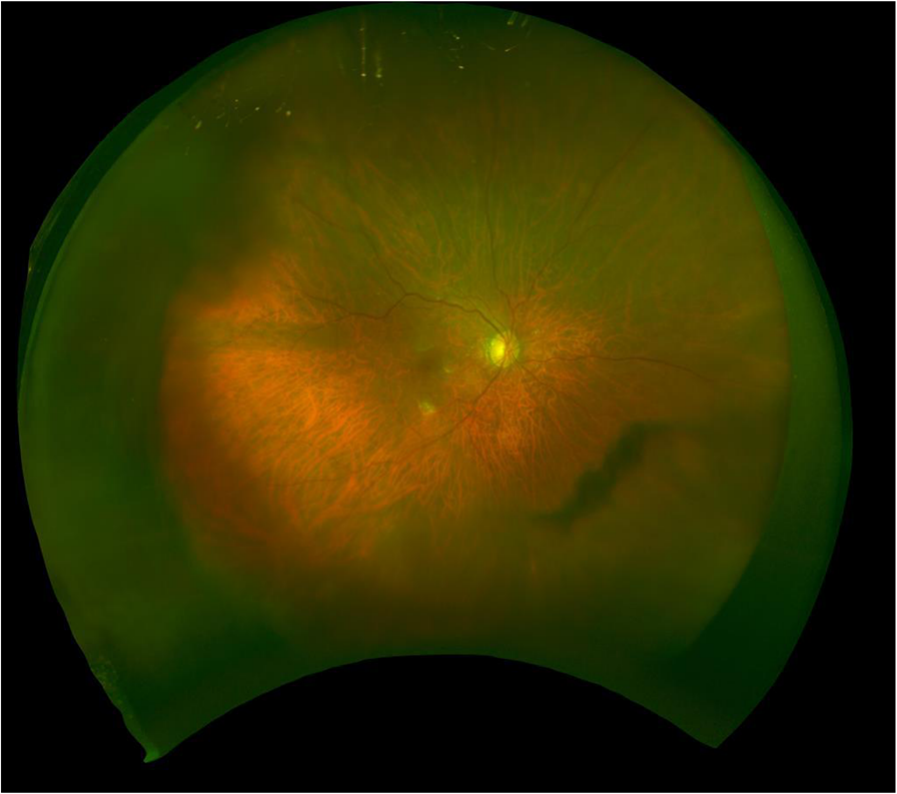
Fundus photographs of the right eye after vitrectomy for three years

Thus, a diagnosis of EBV-associated primary intraocular LPD with no underlying immunodeficiency was made. Therefore, an intravitreal injection of methotrexate (0.4 mg/0.1 ml) was used to treat the ocular inflammation. After the surgery, no inflammations were detected in the anterior chamber and the vitreous of the right eye for 3 years (Fig. 3) and his visual acuity improved to 18/20.

## Discussion and conclusions

EBV is one of the most common viruses infecting over 90% of the adult population worldwide [2]. The virus latently infects circulating B lymphocytes, and this infection is almost always controlled at subclinical levels [2]. However EBV is associated with lymphoproliferative disorders in immunocompromised patients [6], and cases of primary ocular EBV-associated B cell lymphoma have been reported in patients with acquired immunodeficiency syndrome [3], in patients undergoing immunosuppressive therapy for organ transplantation, and patients with rheumatoid arthritis [4, 5].

In our case, there were no systemic diseases leading to immunodeficiency and systemic workup revealed no other sites of lymphoproliferation. EBV can cause chronic infections in individuals without apparent immunodeficiency called chronic active EBV (CAEBV) infections. These infections are characterized by chronic or recurrent infectious mononucleosis-like symptoms and mainly affect children and young adults [7]. Fever, liver dysfunction, splenomegaly, lymphadenopathy, thrombocytopenia, and anemia are the common signs of CAEBV infections [8]. However in our case, there were no infectious mononucleosis-like symptoms and signs, such as fever and cervical lymphadenopathy, and the plasma concentrations of EBV DNA were not detectable. Therefore, we concluded that our patient did not have CAEBV infection.

Similar to the findings in our case, Maruyama and colleagues reported a case of primary intraocular natural killer-T cell lymphoma due to an EBV infection without any systemic diseases [9]. Although the number of reported cases has been small, lymphoma due to EBV infection can develop even without underlying immunodeficiency.

In conclusion, we report our findings in a case of intraocular LPD in an immunocompetent patient with no underlying disease. Anterior aqueous humor PCR and vitreous flow cytometry were helpful for the diagnosis of a primary intraocular EBV-related B-cell lymphoma. This is an aggressive disease that requires systemic treatment to improve the survival, our case was fortunately treated by vitrectomy and an intravitreal injection of methotrexate.

## Data Availability

All data supporting the conclusions of this article are included in this published article.

## Abbreviations

EBV: Epstein-Barr virus
LPD: Lymphoproliferative disorder
BCVA: Best-corrected visual acuity
PCR: Polymerase chain reaction
MRI: Magnetic resonance imaging
FDG-PET/CT: 2-Deoxy-2-[^18^F] fluoro-D-glucose positron emission tomography computed tomography
VCA: Virus capsid antigen
EBNA: EBV-encoded nuclear antigen
CAEBV: Chronic active Epstein-Barr virus
